# Digital Light Processing 3D Printing of Polymer Composites Based on Tunable Curing Resins with Photoswitchable Molecules

**DOI:** 10.1021/acsaenm.5c00401

**Published:** 2025-10-30

**Authors:** Saiful Islam Sagor, Anasheh Khecho, Erina Baynojir Joyee

**Affiliations:** Department of Mechanical Engineering and Engineering Science, University of North Carolina at Charlotte, Charlotte, North Carolina 28223, United States

**Keywords:** digital light processing (DLP), polymer composite, silicon carbide, azobenzene, isomerization

## Abstract

This study presents an additive manufacturing (AM) technique, photoswitchable direct light processing (P-DLP), which utilizes a dynamic mask imaging photoinitiation approach to mitigate light-scattering effects caused by filler particles like silicon carbide (SiC) in composite printing. Vat photopolymerization AM process offers high precision but faces significant challenges in balancing speed and resolution, material instability, and requiring extensive support structures during fabrication. The P-DLP technique overcomes these limitations by employing a dynamic masking system, where ultraviolet (UV) light initiates photopolymerization and visible (blue) light selectively inhibits undesired polymerization. This mechanism allows for precise control over the curing process, enabling the fabrication of complex high-resolution structures while minimizing scattering-induced distortions. A key aspect of this work is the resin formulation incorporating azobenzene as a photoswitchable additive, enhancing the controllability of the polymerization kinetics. UV–vis spectrophotometry results showed that azobenzene extended the absorption spectrum into the blue region, with higher concentrations significantly increasing the absorbance in the 380–500 nm range, confirming its potential as a photoinhibitor. Despite reductions in mechanical properties, the proposed dual-wavelength P-DLP method demonstrated robust control over layer curing, successfully inhibiting unwanted polymerization in the boundary and void regions. This enabled high-resolution printing with minimal overcuring artifacts. The advancements in P-DLP make it well-suited for applications demanding high precision and structural integrity, including optical, medical implants, and soft robotics. Overall, this approach marks a significant advancement in composite AM by overcoming key limitations of conventional methods and enabling the faster, more accurate fabrication of complex components for industrial and biomedical use.

## Graphical abstract

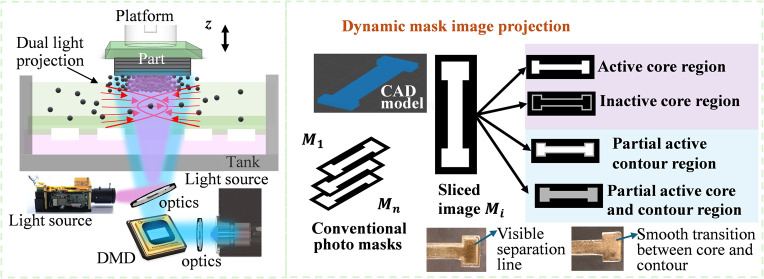

## 1 Introduction

Composite materials have become an essential class of materials in advanced engineering applications due to their capacity to integrate distinct material properties into a single optimized system. Typically composed of a polymer matrix and reinforcing filler particles, composites offer enhanced mechanical, thermal, and functional properties that outperform those of conventional single-phase materials. Key advantages include high strength-to-weight ratio, enhanced durability, and the ability to tailor performance attributes to specific design requirements.^1^ These features make composites especially attractive for high-performance fields such as aerospace, automotive, defense, and increasingly biomedical engineering. In the biomedical domain, composite materials are employed in applications ranging from structural scaffolds and prosthetics to implants and wearable electronics, where their biocompatibility, strength, and chemical resistivity are particularly beneficial.^2–4^

To meet the demand for customized and complex biomedical devices, additive manufacturing (AM) has emerged as a critical fabrication strategy. AM technologies allow for the layer-by-layer construction of patient-specific structures with high geometric complexity and minimal material waste.^5,6^ Among AM techniques, vat photopolymerization (VP) has gained widespread attention for its superior resolution, surface finish, and ability to process intricate geometries. VP process can be divided into two main categories, stereolithography (SLA),^7^ which uses a point-wise scanning laser to cure resin, and digital light processing (DLP),^8^ which projects entire layer patterns via spatially modulated light. Compared with SLA, DLP offers higher throughput and spatial control through digital mask manipulation, making it more suitable for high-speed, precision fabrication.

Researchers have extensively leveraged DLP-based VP for fabricating composite structures by dispersing micro- or nanoscale fillers into photosensitive resins. These reinforcements can significantly improve the mechanical and functional performances of printed parts. However, the addition of other components to pure photosensitive monomers introduces light-scattering issues, affecting the polymerization process. These challenges arise from the complex interactions between light and material, including refraction, scattering, and absorption kinetics.^9^ Filler particles disrupt the uniform transmission of curing light, causing light scattering, refraction, and absorption within the resin. This leads to nonuniform energy distribution, limited curing depth, poor layer adhesion, and defects in the printed geometry. Especially in DLP systems, where entire cross sections are cured simultaneously, the presence of scattering particles can severely impair print fidelity and structural performance. Such inconsistencies can lead to overcuring or undercuring, ultimately impacting the mechanical properties and dimensional accuracy of the final printed part.^10^

To mitigate these issues, researchers have proposed various approaches. Within DLP systems, different manufacturing configurations have been explored to control and project light for curing.^11^ Researchers have explored various solutions, including optimizing exposure parameters and light energy distribution. For mask-based DLP, controlling the exposure time of different pixels helps regulate overcuring by adjusting the light energy distribution in the exposed area.^12^ Additionally, the mask-division method, proposed in,^12^ suggests segmenting light exposure to reduce scattering effects in the boundary region. Digital micromirror device (DMD)-based projectors are widely used in commercial and high-performance research settings due to their ability to reflect high-intensity UV light with excellent spatial resolution.^13^ These systems utilize an array of microscopic mirrors that rapidly tilt to direct light pixel by pixel. Liquid crystal display (LCD)-based DLP systems, by contrast, use a transmissive liquid crystal panel to modulate light in a planar manner.^14^ While LCD-based systems offer a cost-effective alternative, they suffer from limitations in UV light transmission due to the absorption and scattering properties of the liquid crystal material, which can reduce the curing efficiency and resolution. Besides these, researchers have also explored LED-array-based segmented projection systems, where spatially controlled exposure is achieved by illuminating sub-regions sequentially.^15^ Wu et al.^12^ used an LED-based light projection system to reduce the overcuring and scattering artifacts by varying local light intensity. In another study, Zhou et al.^16^ have used a hybrid DLP laser system that combines wide-area projection with precise laser reinforcement for improved interlayer bonding in composites.

Most of the described approaches still rely on material-dependent parameters such as particle size, shape, and refractive index, meaning that any change in filler composition will need re-optimization. This limits the adaptability and real-time control over polymerization. Besides the manufacturing-based configuration of DLP systems, some studies have developed index-matched resin formulations to reduce the refractive mismatch between fillers and the surrounding medium. The use of UV absorbers to localize light penetration has also been explored although this can introduce vertical gradients in curing, affecting the layer uniformity. For example, Xie and He^17^ demonstrated that exceeding 0.5 wt % of graphene oxide in LCD-based VP systems led to significant light attenuation, preventing effective polymerization. Similar limitations have been observed in SLA systems using nonwoven glass or carbon fiber mats, where optical obstruction restricts the feature resolution.

To optimize VP 3D printing for high spatial resolution and defined architectures, a deeper understanding of the molecular structure of photopolymerizable inks and the kinetics of the photopolymerization reaction is required.^18^ Further ink development is necessary to achieve the resolution and speed needed to meet the increasing demand for rapid fabrication and mass production.^15^ A major gap remains in existing strategies that lack the ability to actively and spatially regulate curing behavior in real time. Most methods rely on passive material design or static light modulation, which are often insufficient for compensating the dynamic optical effects introduced by high filler loadings. This constraint motivates the need for a more adaptive and tunable curing mechanism that can respond to complex light–material interactions. One notable effort to overcome such spatial limitations is the Solution Mask Liquid Lithography (SMaLL) process introduced by Dolinski et al.,^19^ which employs a solution-based photochromic mask to form photobleaching fronts and selectively inhibit light penetration. This approach enables one-step multimaterial patterning without physical mask switching and has been successfully demonstrated for effective 3D printing, including soft joints and brick-and-mortar-inspired structures. While highly effective, the method relies on managing additional fluid layers and solution masks, which may sometimes face challenges in integration with existing DLP platforms. In contrast, our method utilizes a dual-wavelength DLP system paired with azobenzene-functionalized resin, where photoinhibition is achieved via molecular isomerization under blue light in the desired location. This enables real-time, programmable spatial control using a DMD-based projection setup without the need for physical masks.

In this study, we propose the use of multiwavelength DLP systems, also known as antagonistic 3D printing,^20^ that incorporate dual-wavelength control, where one wavelength activates polymerization and the other inhibits it. This approach provides real-time spatial control over the curing behavior. This wavelength-specific chemical control represents a significant advancement for printing with light-scattering fillers, offering a more reliable curing depth and structural fidelity in composite materials. The use of voxel-based exposure in DMD systems allows for highly programmable and accurate photopolymerization; however, in composite systems with light-scattering fillers, even the highest-resolution projection may suffer from uneven curing. In such cases, incorporating a dual-wavelength approach provides an innovative solution by enabling not only light-pattern control but also wavelength-specific chemical control of polymerization. This introduces an extra dimension of spatial precision, where polymerization can be selectively activated or inhibited in targeted regions depending on the wavelength applied.

Our study aims to translate this concept into a practical solution by integrating wavelength-specific control directly into the materials and optical system design. Building upon this concept, we propose a materials- and optics-based strategy to realize such control in practice. We developed a novel DLP-based approach utilizing a photoswitchable polymer resin system incorporating azobenzene, which is a well-characterized photochromic molecule. Azobenzene undergoes reversible photoisomerization when exposed to different wavelengths of light, transitioning between trans and cis states.^21^ This molecular switching mechanism can be harnessed to regulate the local polymerization activity through light wavelength selection. In our dual-wavelength DLP platform, UV light serves as a polymerization initiator, while blue light functions as a photoinhibitor. This dual-wavelength control enables spatially resolved, pixel-by-pixel modulation of the curing process, allowing us to counteract the nonuniform curing effects caused by light-scattering fillers such as silicon carbide (SiC).

In this study, we further develop and evaluate a series of azobenzene-functionalized composite inks containing varying concentrations of polymer matrix, SiC, and photoinhibitory components. The optical behavior of these formulations was investigated using UV–vis spectrophotometry to understand how light absorption and transmittance change with filler content. Test structures were fabricated using a custom-built DLP prototype and their mechanical properties were assessed through tensile testing, quantifying the influence of photoinhibition and filler loading. Additionally, a mask-based spatial light modulation technique was implemented and compared with conventional uniform exposure with contour-specific mask designs to enhance boundary resolution and reduce overcuring.

This work introduces a dynamic, wavelength-selective strategy for DLP-based composite printing that addresses the long-standing limitations imposed by optically dense filler systems. By integrating smart resin chemistry with programmable light control, our method provides a versatile and scalable framework for manufacturing high-fidelity, mechanically robust composite structures. The proposed approach holds broad implications for applications in biomedical devices, photonic materials, soft robotics, and precision engineering, where functional composites and geometric complexity are in increasing demand.

## 2 Materials and Methodology

### 2.1 Material Preparation

#### 2.1.1 Ink Material Selection

To test the concept of curing property manipulation described in Section 1, a composite resin was prepared for testing. In this study, for testing the optical properties, seven different ink compositions were prepared by using an acrylic-based photocurable polymer resin (3DM ABS, 3D Materials Inc.) as a base matrix. This resin contains acrylic acid esters, acrylated monomers, oligomers, and TPO-solid (diphenyl­(2,4,6-trimethylbenzoyl)­phosphine oxide) and BAPO (phenylbis­(2,4,6-trimethylbenzoyl)­phosphine oxide) as the photoinitiator.^22^ Acrylate-based photopolymer resins are among the most commonly utilized formulations in different applications, including bone cement, contact lenses, and orthopedic applications.^23^ When exposed to UV light, the photoinitiator absorbs the light energy and produces free radicals, as illustrated in Figure 1(a). These free radicals trigger the polymerization process of the acrylate materials, allowing them to cure within seconds. Along with methacrylate resin, SiC powder (US Nano, Houston, Texas) with an average particle size of 40 μm and azobenzene (with a purity of 98%, Sigma-Aldrich, United States), as a photoswitchable molecule, were mixed in different ratios to prepare the ink resin suspension. The chemical structures of these base components of the prepared ink samples are shown in Figure 1(b). Photoexcitation of the N=N bond in azobenzene molecules under exposure to specific wavelengths of light enables reversible photoisomerization.^24–29^ These characteristics make azobenzene a suitable candidate for the wavelength-selective modulation of the photopolymerization process. The composite inks with varying concentrations of SiC particles and azobenzene were prepared as detailed in Table 1. The selected concentration ranges were determined based on both experimental feasibility and the need to evaluate the effects of each component on light transmission and curing performance. A range of SiC concentrations (0–3 wt %) was tested to assess its influence on optical properties, specifically transmittance and absorbance using UV–vis spectrophotometry. As the SiC concentration increased, the transmittance steadily decreased. At concentrations above 3 wt %, the samples appeared completely black, and spectrophotometric measurements recorded zero transmittance across the UV–visible spectrum, indicating full light blockage. This suggests a saturation effect, where additional SiC no longer influences light transmission in a measurable way due to the extreme optical opacity. For this reason, spectrophotometric analysis beyond 3 wt % was not feasible with our setup, and our study focuses on compositions within the transmissive range (0–3 wt %).

**Figure 1:**
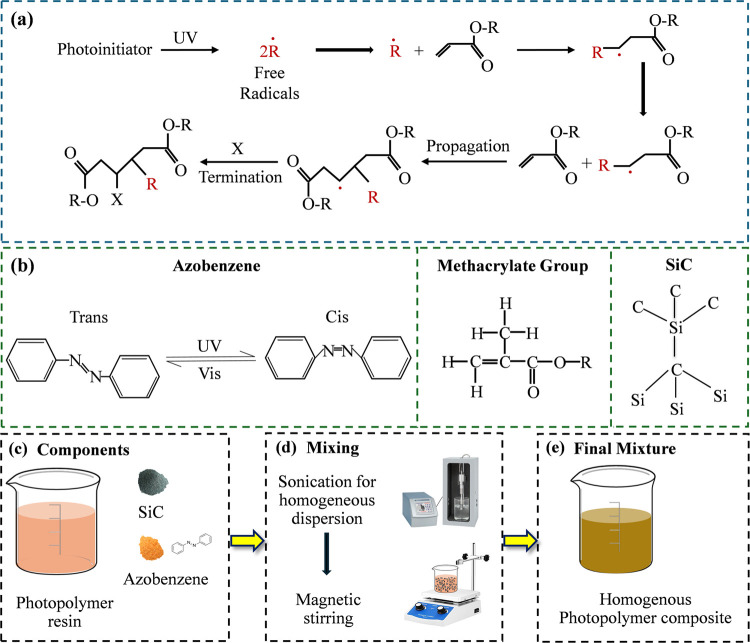
(a) Photopolymerization process of methacrylate-based polymer resin; (b) molecular structure of azobenzene, photopolymer resin, and atomic arrangement of SiC particles; (c–e) preparation steps of the azobenzene-SiC-polymer suspension.

**Table 1:** Composition of the Photopolymer Ink Used in This Study

composite	azobenzene (wt %)	SiC (wt %)	resin (wt %)
C0	0	0	100
C1	0	1	99
C2	0	3	97
C3	1	0	99
C4	3	0	97
C5	0.5	1	98.5
C6	1	3	96

Azobenzene concentrations were varied to evaluate their minimum inhibitory effect on polymerization under dual-wavelength exposure. During this process, it was experimentally found that concentrations above 1 wt % completely suppressed the curing of the composite resin under visible light, even with exposure times extended up to 120 s. These samples remained uncured, likely due to excessive light absorption or strong photoinhibition of azobenzene, which inhibited the formation of reactive radicals needed for polymerization. For this, 1 wt % azobenzene was selected as the maximum usable concentration, balancing inhibition capability with the ability to initiate polymerization under controlled light exposure. An uncured sample after 120 s of exposure has been included in the Supporting Information (Figure S1) to support this observation. The maximum curing depth achieved upon the addition of 1 wt % azobenzene (C3 formulation) was approximately 280 μm. This measurement was conducted by filling the C3 composite into a transparent container and exposing it to projected light for 90 s. After curing, the solidified polymer layer’s thickness was measured from four different locations, with the average yielding a curing depth of 280 μm. In contrast, for the C4 formulation (consisting of 3 wt % azobenzene), no curing occurred after 90 s of exposure, which results in a curing depth close to zero. For all curing tests, the standard deviation of the measured curing depths was approximately ±0.05 mm.

#### 2.1.2 Ink Preparation

Figure 1(c–e) illustrates the preparation of the composite suspension, which followed two sequential steps. First, an ultrasonic sonicator (Qsonica Q700, 700 W, 20 kHz, United States) was used for 10 min to disperse the particles within the resin suspension. The samples were placed in a glass beaker and submerged in chilled water to dissipate heat generated during sonication. The sonicator operated at 20 kHz with a pulse cycle of 5 s to effectively agitate and break up any particle agglomerates. Following sonication, the suspension was mixed using a temperature-controlled magnetic stirrer (Cheshire Enterprise, LLC, United States) at 500 rpm for 5 min. This ensured a uniform distribution of the particles and helped maintain suspension homogeneity during printing.

### 2.2 Spectrophotometric Analysis of Photoswitchable Ink

Spectrophotometry determines how light interacts with matter through reflection, refraction, scattering, absorbance, and transmittance by quantifying the amount of light being absorbed, reflected, or transmitted at a particular wavelength.^30^ Absorbance is a dimensionless quantity that varies from zero for completely transparent samples (where transmittance is 100%) to an infinitely large value for fully opaque samples (where transmittance is 0%).^31^ In this study, the absorbance of the developed inks was examined using a UV–visible spectrometer (Goyojo, China). This spectrophotometer operates at 110 V, 50/60 Hz, with a 6 nm Tungsten Lamp and a wavelength range of 350–1020 nm to determine the optical properties. In a spectrophotometer, absorbance is determined by directing a collimated beam of light at a specific wavelength through a flat, parallel-sided material that is perpendicular to the beam, as illustrated in Figure 2. The intensity of the light entering the sample (*I*_0_) is greater than the intensity of the light that exits (*I*) because some of the energy is absorbed by the molecules within the sample, and these absorbed energy values are shown in the spectrophotometer as absorbance. The relationship of emitted light (*I*_0_) and transmitted light (*I*) with absorbance (*A*) is determined by the following eq 1 ^31^

1 $$A=-\log_{10}\left(\frac{I}{I_{0}}\right)$$

**Figure 2:**
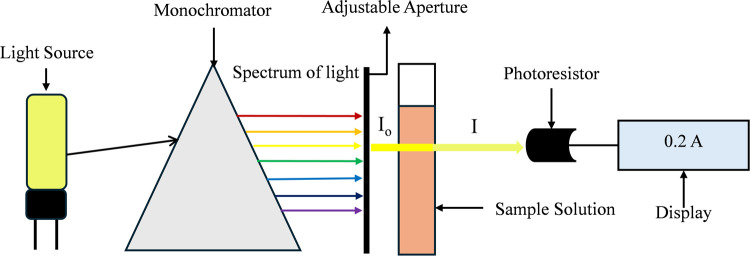
Schematic diagram of spectrophotometry.

In this study, the spectrophotometer was first turned on and allowed to warm up for 30 min. A blank solution of distilled water was then used to calibrate the instrument. After calibration, the samples were placed in 1 cm wide transparent glass cuvettes, wiped with alcohol to eliminate any interference in light transmission, and inserted into the chamber cell. The absorbance values of pure resin and composites with different concentrations were calculated using eq 1 across a wavelength range of 350–700 nm.

Subsequently, absorption coefficients were calculated using Beer–Lambert’s law, which establishes a relationship between wavelength and absorbance. This was done based on the following eq 2 ^32^

2 $$\alpha =2.303\times \frac{A}{d}$$

where α is the absorbance coefficient (cm^–1^), *A* is the absorbance value, and *d* is the thickness of the samples in cm.

### 2.3 DLP Curing with Conventional Mask

A custom DLP 3D printer prototype was developed to test the developed inks. This setup includes an imaging unit, a resin vat with a transparent bottom surface, a linear actuator to lift the build platform, and two linear stages that allow the resin vat to slide. Different parts of the prototype are shown in Figure 3(a). A visible light projector was used as the imaging unit equipped with a 1024 × 768 microarray and an envelope of 42.7 mm × 32 mm, having modified lenses to project images according to the mask over an adjustable working distance. The projection settings, including focus, brightness, and contrast, were adjusted to produce a clear projection image on the transparent plane of the resin vat. The 3D printer was designed with the projector positioned vertically relative to the resin tank, eliminating the need for reflecting mirrors.^33^ This design choice reduces the size of the printer and offers greater flexibility in defining the printing area. A microcontroller was used to move both the vat and platform according to a 2D mask image. To reduce the separation force of the cured part from the tank surface, the bottom of the resin vat was coated with polydimethylsiloxane (PDMS) film due to its inherent property of antisticking and durability.^34^

**Figure 3:**
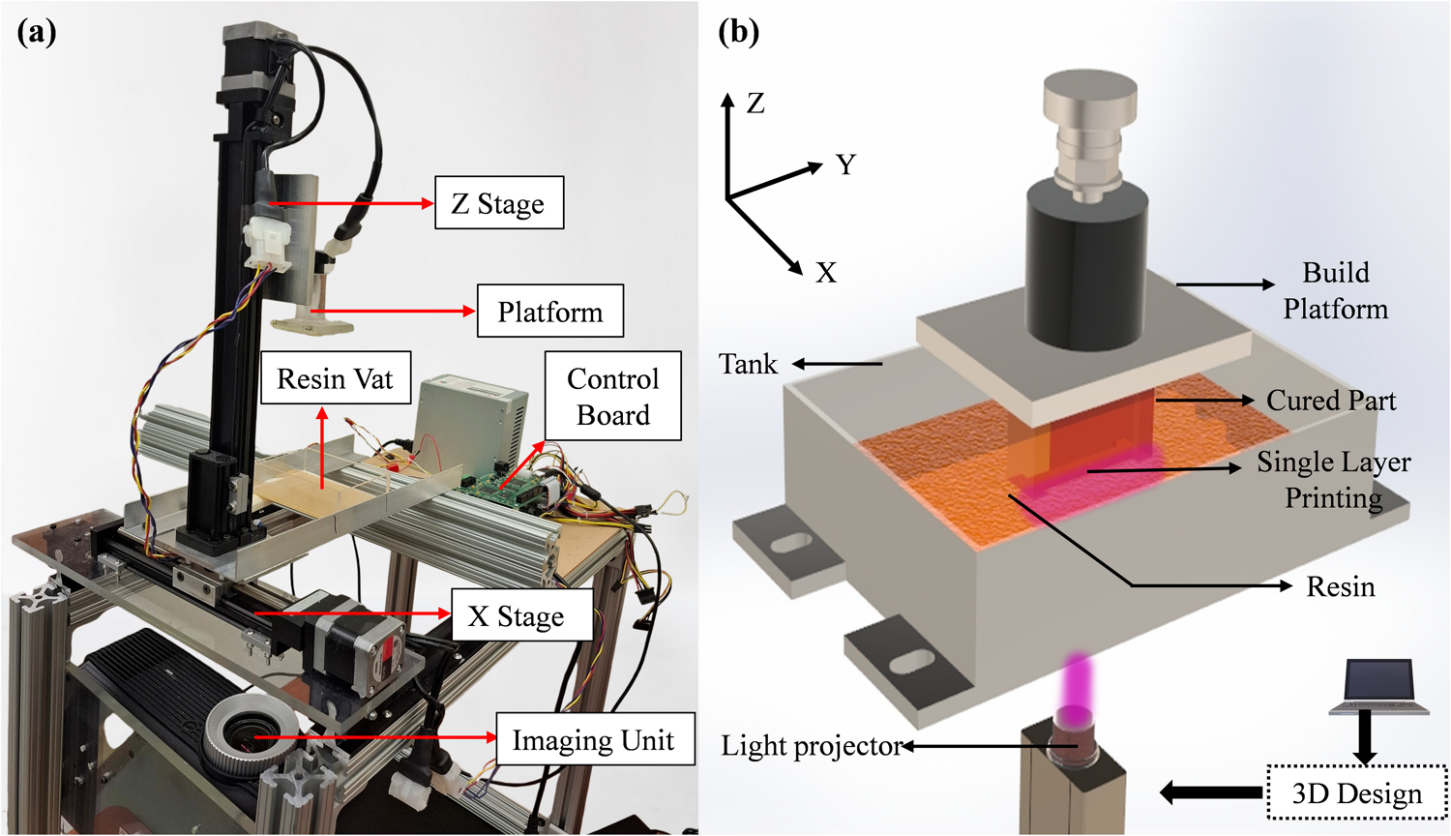
(a) Experimental setup of the DLP prototype; (b) bottom-up layer-by-layer printing process in DLP printing.

A digital mask planning and process control slicing software was developed using C++, as shown in Figure 4(b). This system integrates geometry slicing, image generation, suspension deposition control, and motion coordination. The software generates image masks based on computer-aided design (CAD), as shown in Figure 4(a), and projects them onto the bottom of the build platform through the resin tank.

**Figure 4:**
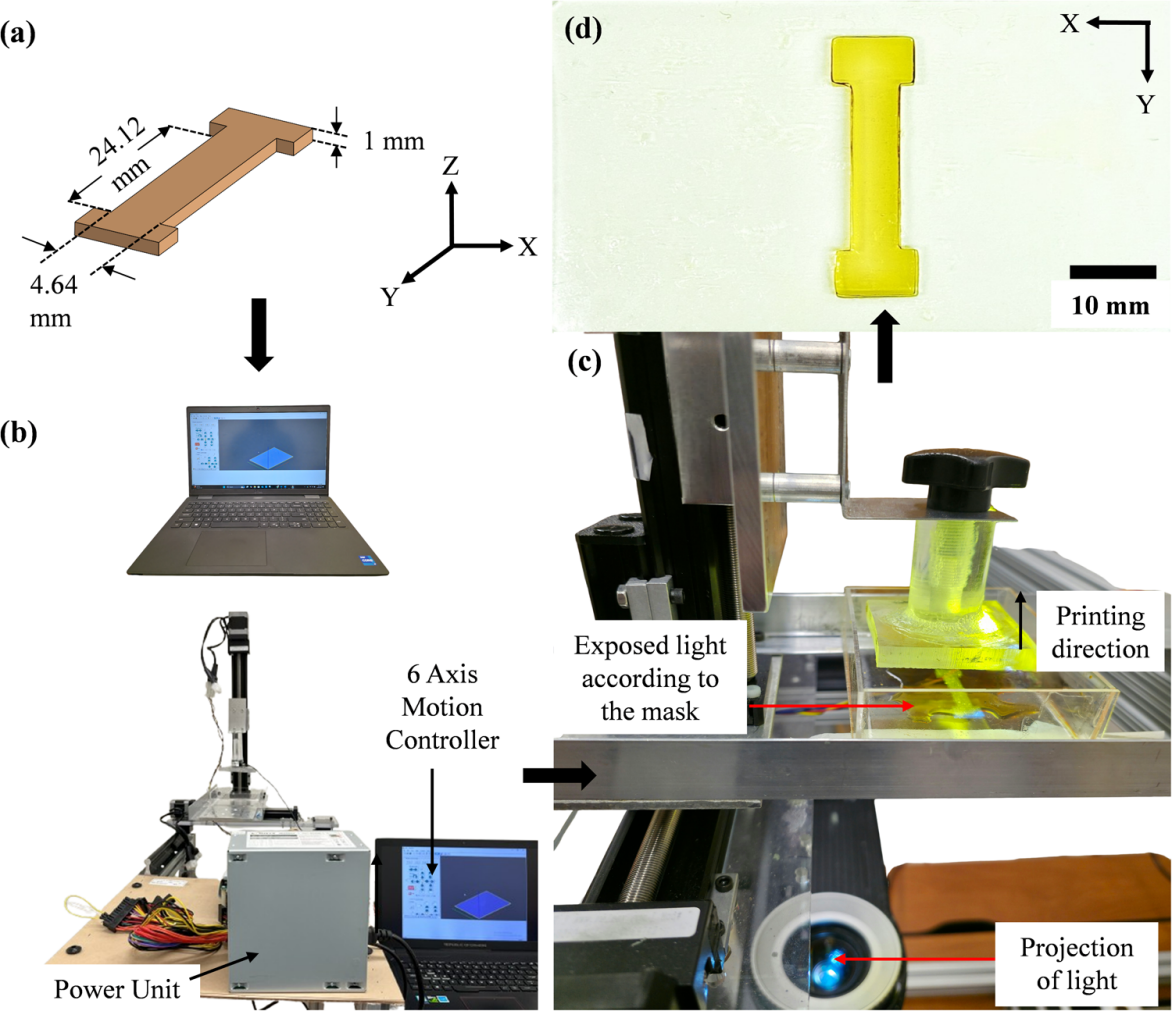
(a) CAD model of the design mask; (b) experimental setup with process software; (c) printing process of the layers; (d) cured part using polymer resin.

At the start of the bottom-up DLP printing process, the build platform lowers into the photopolymerizable liquid resin until it is positioned one layer thickness above the PDMS layer. The layer thickness is controlled by maintaining the distance between the resin vat surface and the build platform. The software converts the CAD drawing into 2D mask images, which are projected from the light source onto the bottom of the building platform through the resin tank. This exposure cures the entire 2D layer, causing the photopolymerized resin to adhere to the build plate and forming the first layer as shown in Figure 3(b). After curing one layer, the build platform moves upward by one layer thickness, which allows uncured resin to refill the space. This process is repeated for the second layer, which adheres to the first layer and continues layer by layer until the complete 3D part is printed. The completed part with three layers, printed with pure resin, is shown in Figure 4(d). These steps were followed to print the same structures (Figure 4a) by using the formulated inks listed in Table 1.

Also, to support dual-wavelength printing, an additional prototype was constructed using two light sources: a DLP UV projector (DLP LightCrafter 4500, 405 nm, Texas Instruments, United States) and an RGB DLP projector (DLP LightCrafter 4500, Texas Instruments, United States). These light sources were mounted orthogonally (90° angle) and combined through a reflective dichroic mirror to direct both UV and blue light onto the resin vat simultaneously, as shown in Figure 10(c).

### 2.4 DLP Curing with Core and Contour Mask

One of the primary limitations of the DLP method for composite printing is the geometric accuracy errors that occur in boundary regions due to light diffraction. As light propagates, it reflects off the digital micromirror device (DMD) that leads to diffraction and an unintended increase in the spot size.^35^ This effect is further exacerbated when printing SiC–polymer composites. The scattering properties of the SiC particles contribute to additional light diffusion. Consequently, this diffracted, unintended exposure extends beyond the target areas, which reduces printing accuracy in the *XY* plane of the fabricated structure. To mitigate these inaccuracies, it is essential to control the curing process at the boundary regions by controlling either the light power intensity or the exposure time. One effective approach to controlling the optical power involves adjusting the grayscale values of the projected mask images.

To investigate the relationship between grayscale values and optical power at the curing position, an experiment was conducted using an optical photodiode power sensor (S120VC, Thorlabs; Figure 5(a)). The optical power sensor was placed between the resin vat and the light source to precisely measure the absolute light intensity at different grayscale values. These absolute intensity values are used to derive the relative light intensity (*I*/*I*_max_), as shown in Figure 5(b).

**Figure 5:**
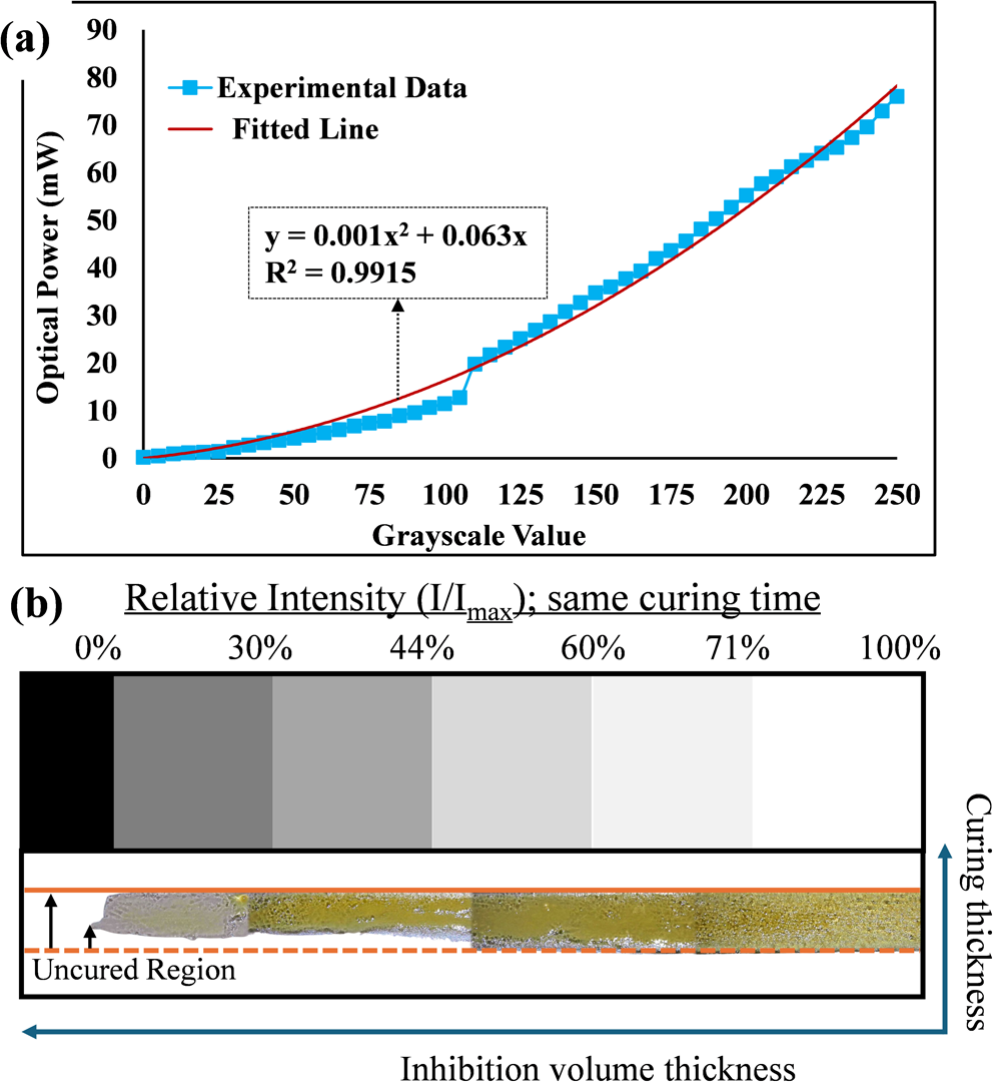
(a) Relationship curve between the grayscale of projected images and optical power of projected light; (b) curing thickness under different relative light intensity.

For each grayscale value, three measurements of optical power were taken to ensure accuracy, and the average values were used in the analysis. The results indicate a quadratic correlation between grayscale values and optical power; as the grayscale value increases, the optical power intensity also increases, and with a grayscale value of 255, it corresponds to the maximum light intensity. Based on the measured data, a second-order polynomial fit was applied, and the following equation was obtained

3 $$P\left(g\right)=0.001\times g^{2}+0.063\times g$$

where *P*(*g*) represents the optical power (mW) and *g* is the grayscale value. An *R* ^2^ value of 0.9915 was obtained from the polynomial regression, which reflects the high accuracy of the nonlinear relationship between the measured optical power and grayscale values. The obtained equation was used for the selection of grayscale levels in the contour mask to achieve partial curing with controlled intensity.

To ensure control over light exposure in boundary regions, the conventional projection masks were divided into two separate masks: i) a core mask and ii) a contour mask. In the first exposure cycle, the core mask was fully activated with a grayscale value of 255, ensuring a maximum light intensity to cure the inner region completely. At the same time, the contour mask was set to a grayscale value of zero, which ensured that the contour region was inactive and prevented the activation of the photoinitiator at the contour region. The exposure time was set to 10 s to ensure that the light with the highest intensity fully cured the core region.

Following the core curing procedure, a second exposure cycle was initiated where the contour mask was partially active with an optimized grayscale value of 180. At this time, the core mask remained deactivated. The optimal grayscale value for the contour region was determined through an iterative optimization approach, leveraging parametric tuning and experimental calibration. A range of grayscale values (150–200) with 2-unit increment was tested, and their effects on boundary integrity and polymerization depth were evaluated. The optimization process employed a gradient-based adjustment strategy, as shown in Figure 6, where the exposure intensity was incrementally modified based on the resulting boundary sharpness and material conversion efficiency. Image processing techniques were used to analyze the cured regions, ensuring uniformity and minimal overcuring effects. A grayscale value of 180 was selected as an optimized value, corresponding to an absolute intensity of 45.64 mW, which results in approximately 60% relative light intensity compared with the maximum intensity of 77.62 mW at the highest grayscale value of 255. This intensity level was found to provide sufficient curing depth while maintaining the dimensional accuracy. At a relative light intensity of 60%, the curing strength was sufficient to create a well-defined rectangular layer, as shown in Figure 5(b). However, residual uncured areas were observed across the cross section, indicating incomplete polymerization. This suggests that while the exposure was sufficient for defining the boundary, internal diffusion limitations or variations in light absorption may have contributed to incomplete cross-linking. When the relative intensity fell below this threshold, curing was insufficient to maintain the intended thickness, leading to edge distortions and discontinuities.

**Figure 6:**
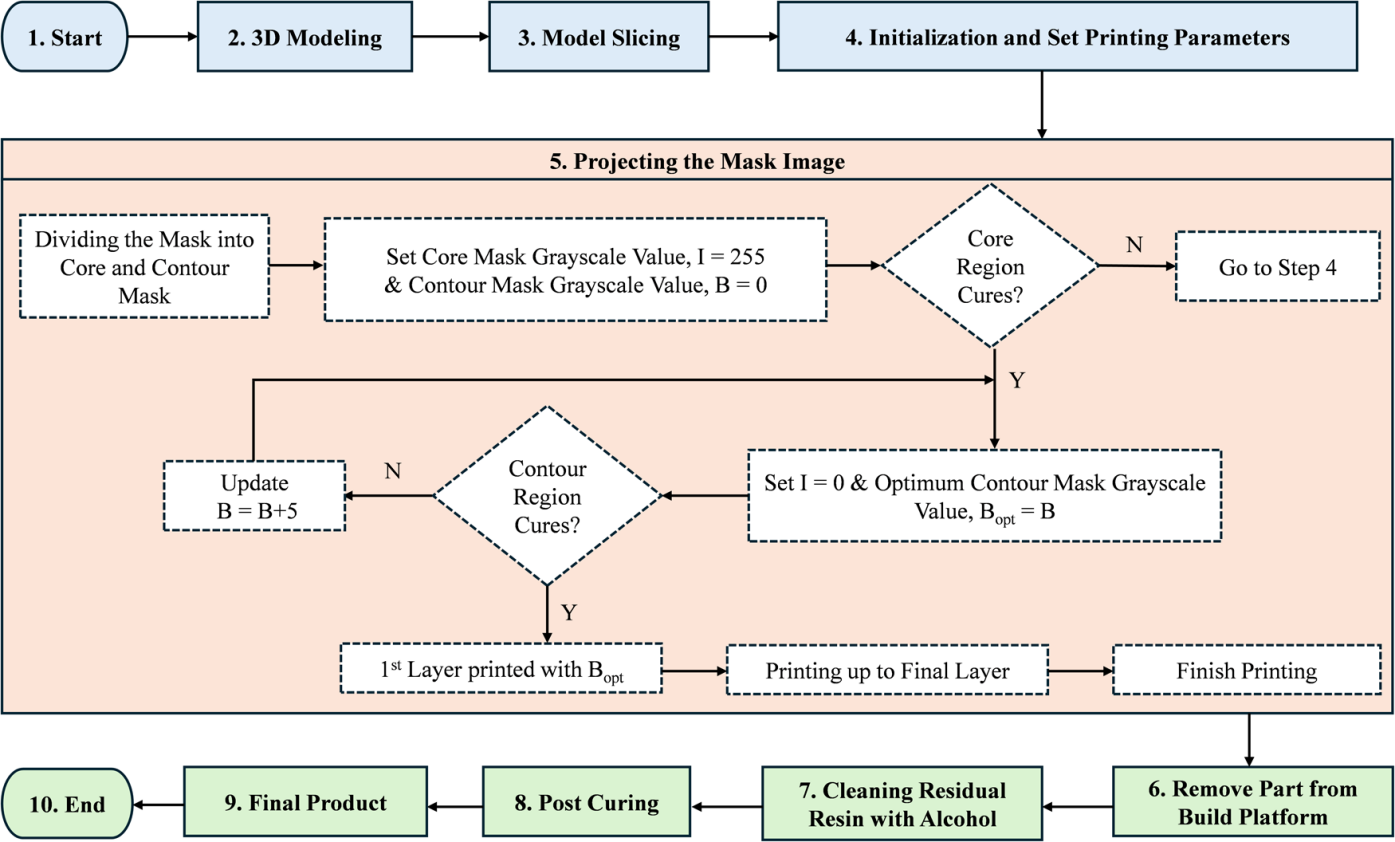
Flowchart of finding the optimum grayscale value of the contour mask.

## 3 Results and Discussion

### 3.1 Spectral Absorption Analysis of Functionalized Inks

Figure 7 illustrates the spectrophotometric behavior of seven different samples prepared and tested to analyze their response at various light wavelengths. Figure 7a shows the absorption characteristics of pure resins (C0) containing TPO and BAPO photoinitiators, which strongly absorb UV light in the 380–405 nm range. For inks C1 and C2 (Figure 7b), adding 1 and 3 wt % SiC increases UV absorbance, though the effect remains small at these concentrations. Figure 7b shows that as the SiC concentration increases, absorbance rises notably in the 350–450 nm range and also extends into a portion of the visible spectrum, particularly for 3 wt % SiC. This suggests that SiC influences light interaction through both absorption and scattering. Since SiC is known for its light-scattering properties and low transparency, higher concentrations of SiC lead to increased scattering and energy dissipation of UV light.^36,37^ As a result, the spectrophotometric measurements show a higher absorbance at elevated SiC concentrations.

**Figure 7:**
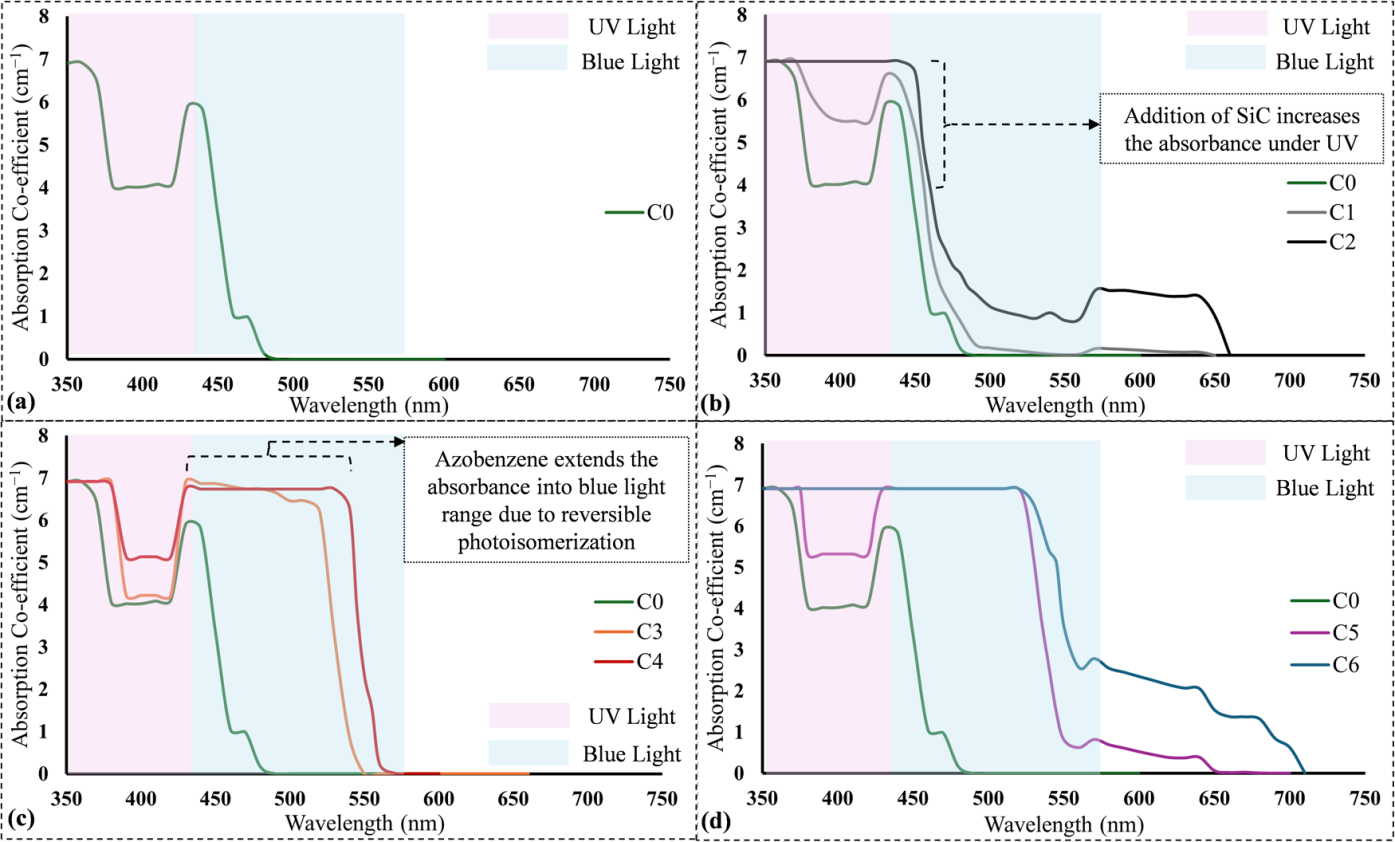
Absorption coefficient spectra of composite formulations across UV and visible wavelength ranges for (a) pure resin; (b) C1 and C2; (c) C3 and C4; (d) C5 and C6.

In the case of inks C3 and C4, the addition of azobenzene extends the absorbance into the blue light spectrum. This is due to photoisomerization, where azobenzene molecules switch between trans and cis forms when exposed to light, enhancing their light absorption ability as shown in Figure 7c.^38^ A comparison between 1 and 3 wt % azobenzene concentrations reveals that absorbance increases near the wavelength of 400 nm, consistent with the Beer–Lambert law. However, in the visible spectrum, a saturation effect is observed at higher concentrations, indicating deviation from the Beer–Lambert law. Additionally, at higher concentrations, a red shift and a plateau in absorbance suggest intermolecular interactions between azobenzene molecules under blue light exposure.^39,40^ This behavior indicates that azobenzene could function as an effective photoinhibitor in ink formulations, improving control over the curing process. For inks C5 and C6 in Figure 7d, which contain both SiC and azobenzene, a combined effect on absorbance is observed. When azobenzene and SiC are present at 0.5 and 1 wt % concentration, respectively, absorbance significantly increases in the 380–530 nm range, likely due to azobenzene’s light-responsive properties.^41^ Increasing the azobenzene and SiC concentration to 1 and 3 wt %, respectively, results in a plateau in absorbance between 380 and 450 nm. This suggests a balance between SiC-induced light scattering and azobenzene absorption. The interaction of these components across the UV and blue spectra suggests that azobenzene absorbs scattered light, particularly near the boundaries of the printed geometry. This property could help mitigate overcuring caused by SiC’s scattering effects, improving the precision of the curing process in DLP printing.

### 3.2 Tensile Testing of Printed Structures

Tensile testing is a destructive mechanical test used to determine key properties such as tensile strength, yield strength, ductility, and elastic modulus of a material. It involves applying a uniaxial tensile force to a specimen until failure to generate stress–strain curve that characterizes the material’s behavior under load. This test is essential for understanding how materials deform and ultimately fail under tensile forces, which is critical for ensuring structural reliability and performance of the composite.^42^

In this study, the samples were prepared following the material preparation procedure described in Section 2.1. Each sample was printed by using the same CAD model from Figure 4(a). The tensile test was performed with a Mark-10 F505-EM test frame, as shown in Figure 8(a). The tests were conducted for each formulation (C0, C1, C2, C5, and C6) using three replicate samples. While Figure 8(b) presents representative stress–strain curves for a single sample to demonstrate mechanical trends, Figure 8(c) summarizes the average ultimate strength from the tests of three printed samples. The printed specimens were carefully secured in the grip of a tensile testing apparatus. The bottom grip remained stationary, while the upper grip applied an increasing tensile load. As the tensile load increased, each sample progressively elongated until it eventually failed. To ensure consistency and reduce experimental errors, three identical samples of each composite were tested. Throughout this process, the force applied and the corresponding deformation were accurately recorded.

**Figure 8:**
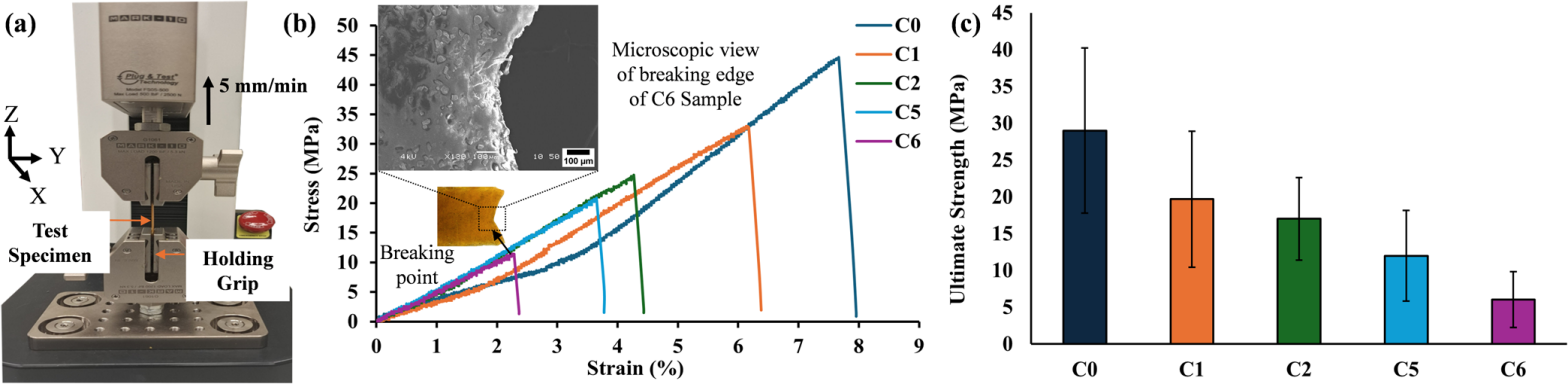
(a) Tensile test setup; (b) stress–strain curves of the printed composite samples (C0, C1, C2, C5, C6); (c) average ultimate tensile strength of each sample group with error bars representing standard deviation.

From Figure 8(b), it is evident that the pure acrylate-based resin (C0) exhibited the highest ultimate tensile strength (∼47 MPa) along with the highest elastic modulus (32 MPa), indicating a well-balanced combination of stiffness and strength. The addition of SiC as a filler had a noticeable impact on the mechanical properties. The C1 composite, containing 1 wt % SiC, showed relatively moderate tensile strength (∼33 MPa) and exhibited a slight reduction in stiffness (30 MPa), suggesting minimal reduction in mechanical performance compared to the C0. In all samples, no clear yield point is observed, and the abrupt drop in tensile stress after the maximum load indicates a brittle failure behavior in the printed composites.

Increasing the SiC content to 3% in the C2 composite led to a significant decrease in both ultimate tensile strength (∼25 MPa) and elastic modulus (19.17 MPa), resulting in more brittleness in the printed part. This degradation can be attributed to the tendency of SiC particles to agglomerate at higher concentrations, which causes nonuniform dispersion within the polymer matrix. Such aggregation forms microclusters that act as stress concentrators and weaken the composite by promoting crack initiation and propagation under load. Consequently, this localized stress concentration undermines the tensile properties of the material.^43^ Moreover, since SiC is an inherently brittle ceramic, its incorporation with polymer matrix exacerbates brittleness and compromises mechanical performance.^44^ The use of different stabilizing agents can improve homogeneity and ensure the uniform distribution of SiC particles within the polymer matrix. Dispersants such as poly­(ethylene imine) (PEI) and silane coupling agents have demonstrated the ability to enhance colloidal stability and interfacial bonding of SiC particles. These agents function through mechanisms such as electrostatic repulsion, steric hindrance, or covalent surface modification, which help to reduce particle clustering and improve stress transfer across the matrix.^45–47^

Furthermore, the incorporation of azobenzene in addition to SiC fillers negatively influenced the mechanical characteristics of the composites. The C5 composite, comprising 0.5 wt % azobenzene and 1 wt % SiC, demonstrated lower tensile strength (∼21 MPa) and a reduced elastic modulus (18.55 MPa). The samples were brittle, and they had undergone up to 4 wt % of strain before breaking. The effect was more pronounced in the C6 composite, which contained 1 wt % azobenzene and 3 wt % SiC, resulting in the poorest mechanical performance among all tested samples, exhibiting the lowest ultimate tensile strength (∼12 MPa) and a comparable elastic modulus (19.99 MPa). The microscopic image of the C6 sample’s fractured surface was taken using a scanning electron microscope (SEM, JEOLJSM-6480 CO., Ltd., Japan), and it shows a rough, irregular edge, indicating a brittle fracture mode, supporting the observation of poor ductility and early failure. The reversible photoisomerization of azobenzene molecules upon UV and visible light exposure alters the molecular geometry from a linear (trans) to a bent (cis) configuration, inducing local disruptions within the polymer matrix. This conjugated structure hinders the mobility of polymer chains, reduces the material’s ability to deform under tensile stress, and contributes to decreased tensile strength and elongation.^48^ Therefore, to preserve the mechanical integrity of such composite systems, the concentration of azobenzene must be carefully controlled to balance its functional benefits with its structural performance. The resulting average ultimate strength with individual standard deviations for each formulation, shown in Figure 8(c), follows the same trend as the representative single-sample stress–strain curves in Figure 8(b), reinforcing the reliability of the results.

### 3.3 Evaluation of Masking Segmentation in DLP Printing

After the optical and mechanical performances of the formulated inks were evaluated, printing tests were conducted to further assess their processability. To test the effectiveness of the proposed masking segmentation technique, a dog bone-inspired 3D model was designed, as illustrated in Figure 4(a). A single layer was cured with the C0 formulation, as shown in Figure 9­(a-b), using the segmented mask projection method outlined in Section 2.4.

It was observed that (Figure 9e) the cured part exhibited an unintended separation line between the core and contour regions, indicating a weaker interfacial bond that compromised the structural integrity of the printed component. This issue arose due to the curing sequence used in the segmented mask projection process. In the initial exposure cycle, as shown in Figure 9(c), the core region was fully activated at a maximum light intensity, while the contour region remained completely inactive. In the subsequent exposure cycle, the contour zone was partially exposed using the optimized grayscale value of 180, found from the approach described in Figure 6, whereas the core region was not re-exposed. Since these regions were polymerized independently under distinct light exposure conditions, the linker monomers at the interface may not have undergone complete cross-linking, which leads to poor adhesion and the formation of the visible separation line. These findings highlight the need for further optimization of the segmentation strategy to ensure uniform polymerization and to enhance interfacial bonding.

**Figure 9:**
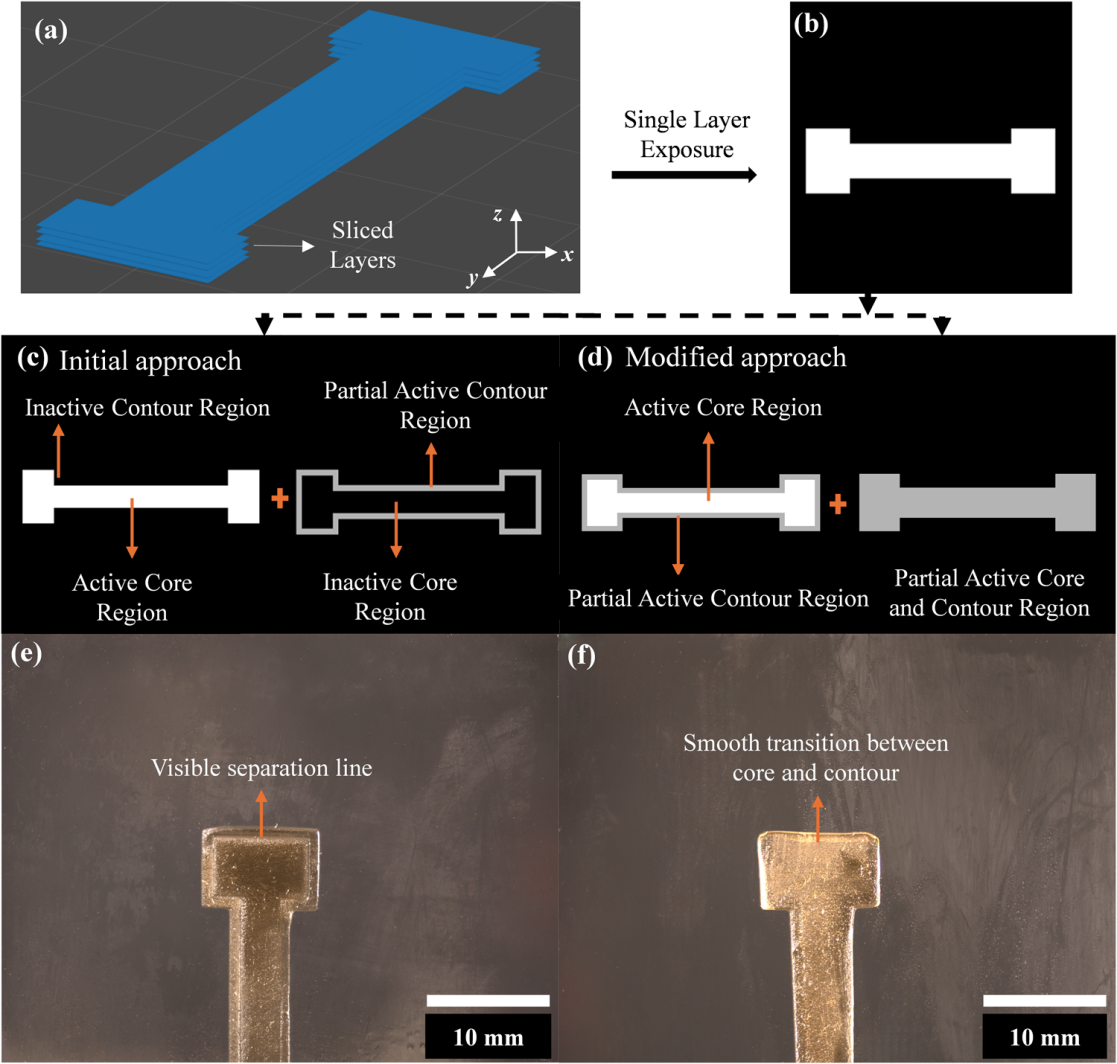
(a) Sliced layers of the CAD model used for mask generation; (b) conventional single-layer mask projection; (c) initial segmented mask approach showing separate activation of core and contour regions; (d) modified segmentation strategy with simultaneous partial activation of both core and contour; (e) printed part from the initial approach showing a visible separation line between core and contour; (f) printed part from the modified approach showing a smooth transition and improved interfacial bonding.

To address this issue, an alternative masking segmentation approach was implemented, shown in Figure 9(d). In this modified method, both the contour and core regions were exposed simultaneously but at different light intensities. During the first exposure cycle, the core region was fully activated at maximum intensity, while the contour region was partially activated at 60% intensity, instead of remaining inactive, as in the previous approach. In the second exposure cycle, the core region was not completely deactivated but instead was maintained in a partially active state with an optimized grayscale value of 180, similar to the contour region.

This simultaneous curing strategy resulted in a more uniform polymerization process, reducing the chance of insufficient cross-linking at the interface. Additionally, to mitigate the risk of overcuring, the exposure time per cycle was reduced to 5 s, which is half the duration used in the initial approach. As shown in Figure 9(f), the modified approach eliminated the separation line, indicating improved interfacial adhesion and structural integrity. However, a slight overcuring effect was observed in the top-left portion of the sample, suggesting that further optimization of the exposure time may be necessary to refine the process and achieve optimal geometric precision.

### 3.4 Concept of Dual-Light DLP Printing with Azobenzene Composite

To evaluate the feasibility of the proposed dual-wavelength P-DLP printing method using photoswitchable composite resins (C4), experiments were conducted using the custom dual-light prototype, described in Section 2.3. Composite samples (C4) were exposed separately to UV and blue light. For both tests, a simple 17 mm × 17 mm square-shaped 2D mask was projected, and a single layer was cured with an exposure time of 10 s. Under UV exposure, significant overcuring beyond the intended masked region was observed due to the scattered UV light (Figure 10a). In contrast, exposure to blue light under the same conditions resulted in no visible polymerization (Figure 10b), which demonstrated the effective photoinhibitory effect of azobenzene.

**Figure 10:**
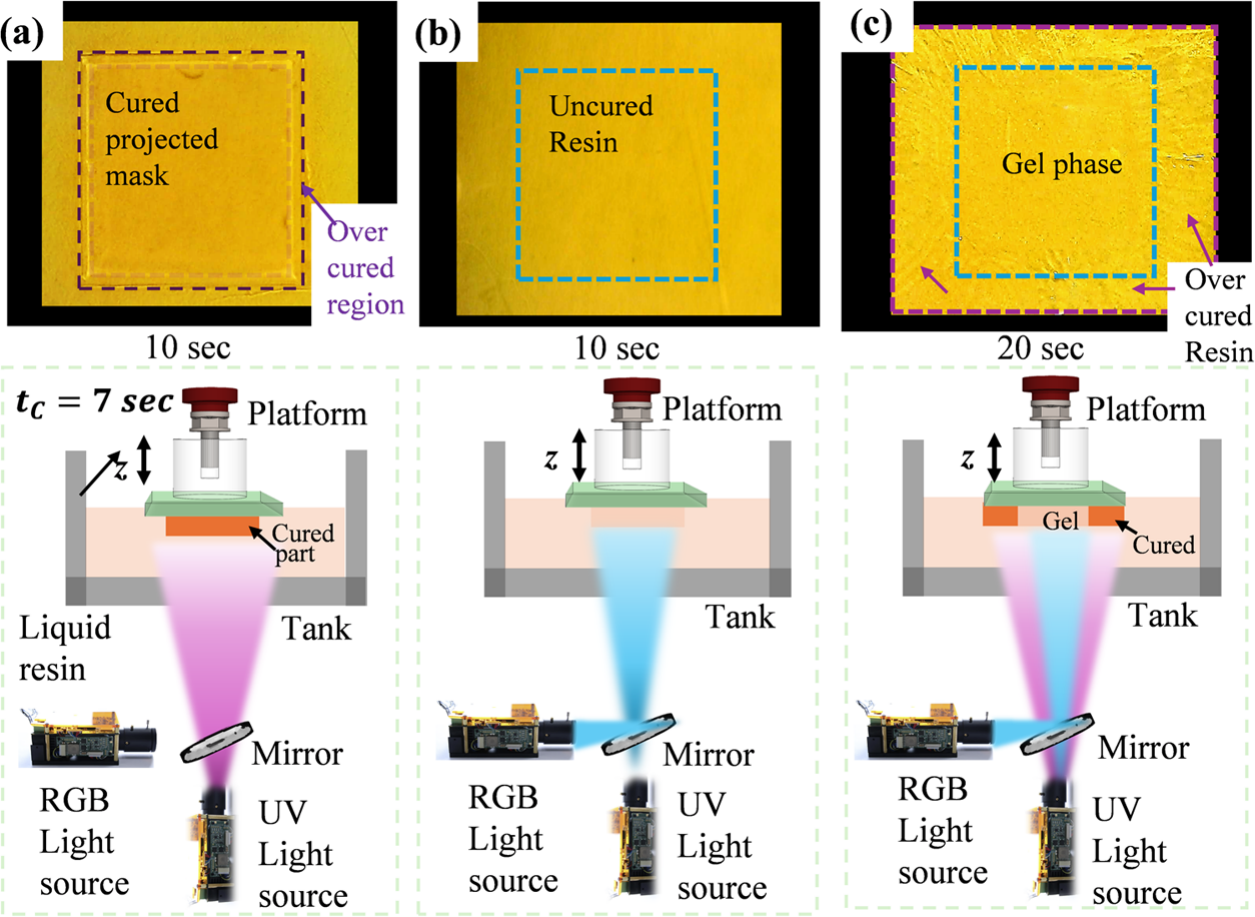
Investigation of photopolymerization of C4: (a) under UV light; (b) under blue light; (c) proposed mask projection system during dual-light DLP printing.

Next, the dual-light printing experiment was conducted using the simultaneous projection of UV and blue light. The exposure time was extended to 20 s. As shown in Figure 10c, the area exposed to UV light showed signs of overcuring, while the region marked with a blue dotted line in the center remained in a gel-like, under-cured state. This indicated delayed or inhibited polymerization under blue light in that region despite the extended exposure, validating the concept of spatially selective photoinhibition using azobenzene under blue light.

Based on these observations, a single layer of thin-wall cylindrical structure featuring multiple internal voids, inspired by the structure of the lotus root (Figure 11a), was cured using the dual-wavelength DLP prototype to demonstrate the feasibility of the proposed P-DLP process. C6 formulation, which includes both SiC and azobenzene, was used to print the lotus root structure. This structure was chosen to examine whether our proposed P-DLP strategy could effectively control polymerization in void-rich, complex geometries by leveraging the photoinhibition effect of azobenzene. The developed prototype currently shows some alignment issues between the UV and blue light sources (Figure 11b). Specifically, in certain areas, the blocking mask projected by the blue light source does not completely overlap with the contour mask from the UV source. The misalignment between the UV contour mask and the blue blocking mask was quantified by comparing the printed structures with the reference CAD design (Figure 11a). First, high-resolution microscopy images of the cured samples were captured (Figure 11e). These images were processed using an overlay analysis approach, where the CAD outline was digitally superimposed onto the printed geometry. Image-processing software (e.g., ImageJ) was used to measure the positional deviation of the printed void boundaries relative to the CAD-defined edges. The misaligned light zones, highlighted in Figure 11e (left), showed an average offset of approximately 1.5 mm, which we attribute to the lateral shift between the two projected masks. In contrast, the aligned light zone (Figure 11e, right) closely matched the CAD geometry, confirming the effectiveness of the proper alignment which allows precise exposure of the blocking mask on both the peripheral and internal void regions. This mask projection approach involved two sequential primary exposure steps. In the first, UV light was used to selectively cure the core and contour regions at optimized intensities, following a segmentation method similar to that described in Section 3.3. Specifically, in the first step, full-intensity UV light was applied to the core, while reduced intensity was used for the contour. In the second step, both regions received partial-intensity UV exposure at optimized gray-scale values to further refine polymerization, as shown in Figure 11(c). Simultaneously, secondary exposure with blue light was applied using a blocking mask positioned around the outer boundary of the UV-cured region and along the internal voids (Figure 11d).

**Figure 11:**
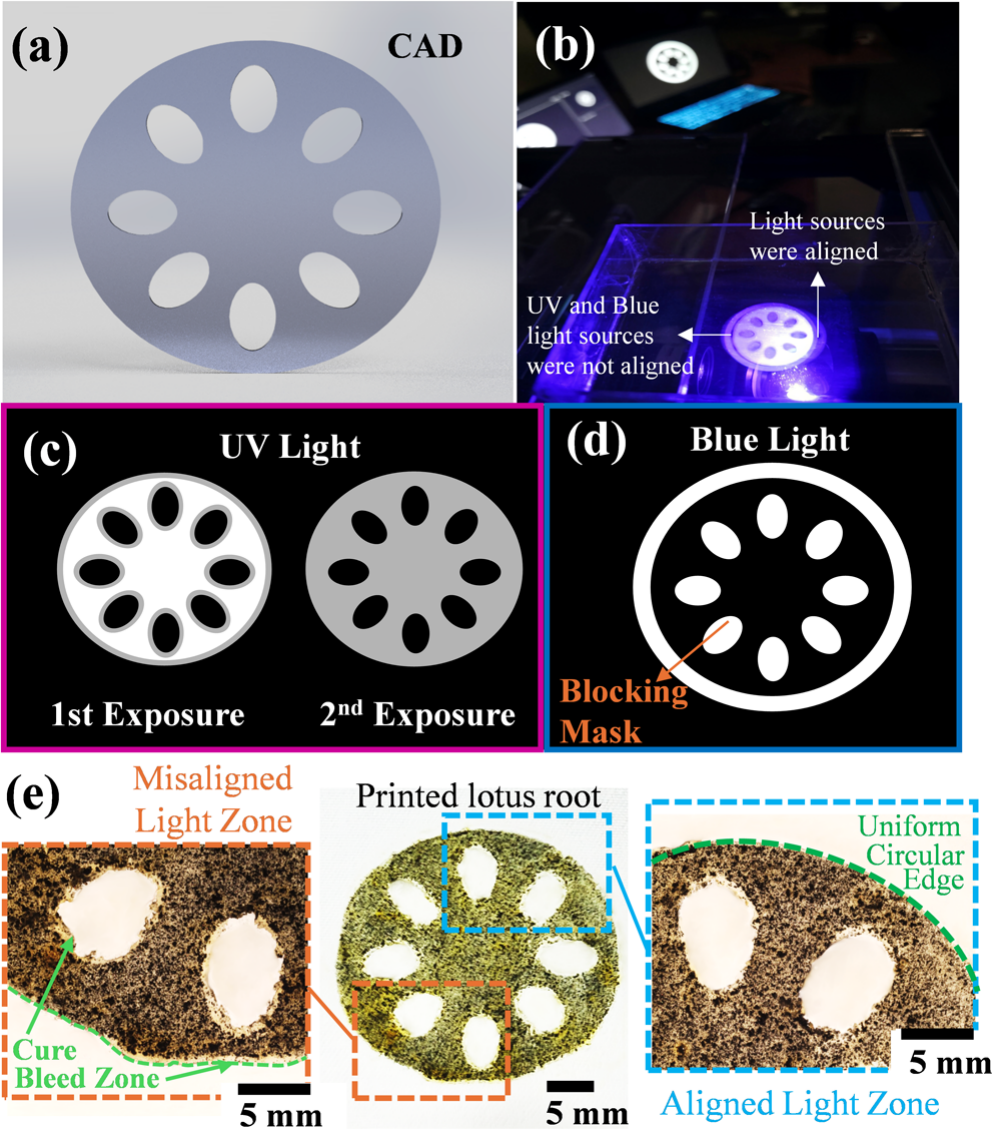
(a) CAD model of the lotus root-inspired structure; (b) light source setup of the dual-wavelength DLP system, showing aligned vs misaligned regions of UV and blue light sources; (c) sequential segmented exposures under UV light for core and contour regions; (d) blue light projection using a blocking mask for photoinhibition in targeted zones; (e) optical image of the printed structure (C6) showing cure bleed regions due to light misalignment (left) and a uniform edge resulting from proper alignment (right), highlighting the effect on internal void preservation.

Figure 11(e) presents a successfully cured layer printed by using the proposed dual-wavelength DLP approach. As shown in Figure 11(e, right), the circular portion maintained a well-defined shape on the side where the blocking mask and the UV contour mask aligned correctly. Successful inhibition was observed in the voids and peripheral boundary areas, where blue light was projected in conjunction with azobenzene. These regions remained uncured throughout the process, validating the effectiveness of photoinhibition in preventing unintended polymerization near UV-exposed zones. The preserved voids, along with well-defined circular boundaries, demonstrate that the inhibition mechanism provided spatial control overcuring and minimized feature distortion. In contrast, on the misaligned side of the sample, blue light did not cover a narrow gap between the UV contour mask and the blue blocking mask. This omission allowed scattered UV light intensified by the light-scattering SiC fillers to reach the resin in that gap, resulting in unintended polymerization. The consequence was visible as a cure bleed zone and a distorted pore edge, as shown in Figure 11(e, left). These visual cues clearly differentiate between successfully inhibited and overcured regions, highlighting the critical role of spatial mask alignment in dual-wavelength photopolymerization systems. Also, it is observed that the regions intended to remain void were successfully protected and preserved in their uncured state, maintaining the designed porous geometry. This demonstrates the printability of our proposed method by the integration of this dual-wavelength strategy shown in Figure 10(c), emphasizing its spatial precision and ability to minimize scattering-induced curing errors common to conventional DLP processes. However, the current implementation requires manual alignment of the dual masks, which poses challenges for scalability and consistent performance. Misalignment between the UV and blue-light masks can lead to curing artifacts at the boundary regions. To address this, future work will focus on developing an automated image registration and alignment algorithm, enabling real-time synchronization of pixel positions between the two projection systems. Additionally, a pixel-wise energy blending algorithm will be implemented to dynamically adjust light intensity based on spatial irradiance profiles, ensuring uniform inhibition and exposure. These improvements aim to enhance the automation and scalability of the proposed dual-mask strategy for broader adoption in high-throughput DLP systems.

## 4 Conclusions

In summary, we demonstrated the feasibility of effectively controlling DLP 3D printing through the incorporation of azobenzene composites in the functional ink, utilizing azobenzene’s photoinhibitory properties under blue light. Through comprehensive spectral absorption analysis and printing experiments, the photoinhibitory capability of azobenzene within functionalized inks was successfully validated. Additionally, this research addressed critical challenges associated with mask DLP 3D printing, such as visible separation lines on printed surfaces, through optimized light-intensity-controlled exposure in the core and contour region. Also, a novel blocking mask concept using the photoinhibitory effect of azobenzene under blue light in dual-wavelength DLP printing is proposed and applied for reducing issues related to overcuring. Our future research will further investigate the practical feasibility of dual-wavelength printing with a blocking mask by properly aligning the blocking mask with the core and contour mask, optimizing the azobenzene concentration, exposure parameters, and enhancing resin formulations to facilitate the fabrication of precise composite structures with enhanced mechanical strength for diverse industrial and biomedical applications.

## Supplementary Material



## Data Availability

The data generated and analyzed during the study are available on request.

## Notes

Schematic illustration (left) and experimental observation (right) of the curing behavior of the C4 composite (3 wt % azobenzene) under 120 seconds of visible light exposure (Figure S1) (PDF)
