# Supplementary material for: Digital Light Processing 3D Printing of Polymer Composites Based on Tunable Curing Resins with Photoswitchable Molecules
Source: ACS Appl Eng Mater. 2025 Oct 30;3(11):3751–63. doi: 10.1021/acsaenm.5c00401 (PMC12670390; doi:10.1021/acsaenm.5c00401)
Supplement: Supplementary file 1 [file em5c00401_si_001.pdf]

Supplementary Figures:

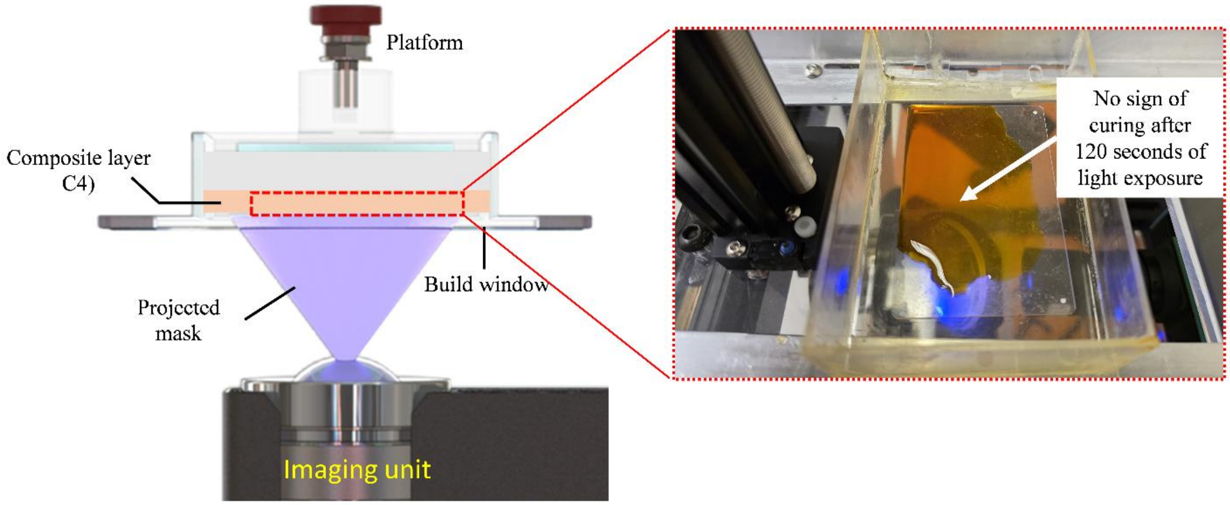

Figure S1. Schematic illustration (left) and experimental observation (right) of the curing behavior of the C4 composite (3 wt% azobenzene) under 120 seconds of visible light exposure.
